# Preoxygenation strategies before intubation in patients with acute hypoxic respiratory failure: a network meta-analysis

**DOI:** 10.3389/fmed.2025.1532911

**Published:** 2025-02-10

**Authors:** Na Ye, Chen Wei, Jiaxiang Deng, Yingying Wang, Hongwen Xie

**Affiliations:** ^1^Department of Nursing, Fourth Affiliated Hospital of Jiangsu University, Zhenjiang, China; ^2^Department of Emergency, Yijishan Hospital of Wannan Medical College, Wuhu, China

**Keywords:** acute hypoxic respiratory failure, preoxygenation, high flow nasal cannula, non-invasive ventilation, network meta-analysis

## Abstract

**Background:**

Patients with acute hypoxic respiratory failure (AHRF) face life-threatening complications during tracheal intubation. Preoxygenation can enhance oxygen reserves and mitigate hypoxemia risk, but the optimal strategy remains unclear. This study aimed to identify the best preoxygenation strategy for these patients.

**Methods:**

We conducted a network meta-analysis of studies published up to July 2024, evaluating conventional oxygen therapy (COT), high-flow nasal cannula (HFNC), non-invasive ventilation (NIV), and their combinations prior to intubation. Data were extracted and analyzed using pairwise and network meta-analysis within a Bayesian framework. Model selection was based on the deviance information criterion (DIC).

**Results:**

A total of 11 randomized controlled trials involving 2,874 patients were included. NIV preoxygenation significantly reduced the likelihood of SpO_2_ <80% during intubation compared to COT (RR 0.28, 95% CI 0.070–0.71). No significant differences were found in lowest SpO_2_, complications, ICU length of stay, or mortality across preoxygenation strategies. HFNC was the most effective for reducing complications, while HFNC combined with COT or NIV showed similar effects on the lowest SpO_2_ during intubation.

**Conclusion:**

Preoxygenation with HFNC appears to be the safest and most effective approach prior to intubation in patients with AHRF compared to other strategies.

**Systematic review registration:**

PROSPERO (CRD42024565053).

## Background

Acute hypoxic respiratory failure (AHRF) occurs when inadequate oxygen supply, due to internal physiological factors, impairs respiratory function, lowers blood oxygen levels, and causes tissue hypoxia, ultimately leading to respiratory failure. AHRF patients often require endotracheal intubation (ETI) and mechanical ventilation, but this carries a significant risk of complications; approximately 25% experience severe desaturation (SpO_2_ <80%) during the procedure (1, 2). Preoxygenation before intubation is crucial for increasing oxygen reserves and delaying desaturation (3, 4), as it prolongs the safe apnea time (SpO_2_ 88–90%) by replacing lung nitrogen with oxygen (5). Safe apnea time is defined as the time from respiratory arrest to a drop in peripheral arterial oxygen saturation (SpO_2_) to 90% (6). Adequate preoxygenation mitigates poor outcomes during apnea by preventing hypoxia and allowing sufficient time for successful intubation. Inhalation of high concentrations of oxygen during intubation attempts further enhances oxygen reserves and may prolong the safe apnea time in awake or cooperative patients. Finally, preoxygenation can reduce complications associated with intubation, such as arrhythmias or respiratory failure.

Current preoxygenation methods include valved masks, high-flow nasal cannula (HFNC), non-invasive ventilation (NIV), and combinations of these methods, and conventional oxygen therapy (COT). HFNC, a non-invasive technique delivering heated and humidified, high-flow oxygen via nasal cannula, effectively corrects hypoxia and reduces hypoxic respiratory failure (7). Compared to NIV, HFNC offers advantages such as increased inhaled oxygen concentration via CO_2_ washout and provision of a heated and humidified environment (8). In contrast, NIV may be poorly tolerated and associated with complications including dry mouth, facial pressure injuries, abdominal distension, and pneumothorax (9, 10). Previous paired meta-analyses have investigated preoxygenation strategies. Song et al. (11) demonstrated the superiority of HFNC to COT in improving oxygenation and prolonging safe apnea during anesthesia induction. Another meta-analysis of 17 randomized controlled trials (RCTs) showed that NIV reduced intubation rates in AHRF patients compared to COT and HFNC, but with variable results and no significant advantage in other outcomes (12). Recent AHRF preoxygenation RCTs necessitate a network meta-analysis (NMA), a method increasingly used to compare multiple treatments (13), to assess the efficacy and safety of various preoxygenation modalities in AHRF patients.

## Methods

### Search strategy

A literature search of PubMed, Web of Science, Cochrane Library, and Embase (up to July 2024) was conducted using the following MeSH terms: ((“Respiratory Insufficiency” OR “Respiratory Failure” OR “Hypoxemic Respiratory Failure” OR “Hypoxemic Acute Respiratory Failure”) AND (“cannula” OR “high flow nasal cannula” OR “HFNC” OR “HFNO” OR “high flow nasal oxygenation” OR “NonInvasive Ventilation” OR “Noninvasive” OR “Non-Invasive” OR “Non-Invasive Ventilation”)) AND (“Oxygenators” OR “preoxygenation” OR “pre-oxygenation”) AND (“Randomised Controlled Trial”)). No language restrictions were applied. This systematic review is registered with PROSPERO (CRD42024565053). Institutional review board approval was not required as only previously published data were analyzed, without the inclusion of individual patient data.

Studies were included if they met the following criteria: (1) data on patients in the study population who underwent tracheal intubation with a diagnosis of AHRF, defined as a respiratory rate higher than 30/min, a FiO_2_ requirement of 50% or more to achieve at least 90% SpO_2_ (or where it was not possible to achieve more than 85% SpO_2_) (14), and an estimated arterial partial pressure of oxygen (PaO_2_)/FiO_2_ ratio of less than 300 mmHg within 4 h prior to enrollment (15); (2) interventions involving various modalities of preoxygenation during tracheal intubation; (3) RCT study design; and (4) outcome metrics including the incidence of post-intubation SpO_2_ <80%, lowest SpO_2_ during intubation, post-intubation-related complications, ICU length of stay (ICU LOS), and ICU mortality. The following were excluded: (1) literature such as reviews, conferences, and case reports; (2) studies with incomplete extracted data; and (3) studies where the full text was not available.

### Study selection and data extraction

Based on the inclusion and exclusion criteria, two reviewers trained in evidence-based nursing reviewed and assessed the relevant literature, collected and categorized the study data, and performed comparative verification. In cases of discrepancies, the reviewers discussed the issues or referred them to a third party for adjudication.

### Risk of bias

RCT quality was assessed using the *Cochrane Handbook for Systematic Reviews of Interventions*, evaluating seven domains: random sequence generation, allocation concealment, blinding of participants and personnel, blinding of outcome assessment, completeness of outcome data, selective reporting, and other biases. The risk of bias for each RCT was categorized as high, low, or unclear using the Cochrane Collaboration tool.

### Statistical analyses

A series of paired meta-analyses were conducted using random effects modeling to evaluate direct associations between interventions and study outcomes. NMA was performed within a Bayesian framework using JAGS (version 4.3.0), R software (version 4.4.0), and the rjags and gemtc packages. To maximize accuracy and efficacy in this analysis, the mean difference (MD) and 95% confidence intervals (CIs) were used to assess the impact of each preoxygenation strategy on the lowest SpO_2_ and length of stay in the ICU in the context of AHRF (16).

Differences were considered statistically significant when the range of 95% confidence intervals did not include 0. The outcomes SpO_2_ <80%, post-intubation-related complications, and ICU mortality were evaluated using relative risk (RR) and 95% confidence intervals, with differences deemed statistically significant when the range did not include 1. The deviance information criterion (DIC) and potential scale reduction factor (PSRF) were also calculated. DIC is widely used for Bayesian model selection, with a smaller DIC indicating a better model fit. A PSRF close to 1 implies good convergence, suggesting the robustness of the consistent model. Model selection was based on the Dias guide for evaluating linear models (17). The dbar denotes the posterior mean of the residual bias, pD denotes the effective number of parameters (leverage), and DIC denotes the deviance information criterion. Smaller dbar values indicate better model fit, and typically, the model with the lowest DIC is chosen to account for model complexity. Differences between models of less than 3 to 5 were considered insignificant (18). The probability of each preoxygenation modality being optimal was determined by assessing rank probabilities, with a higher probability of reaching rank 1 indicating a higher probability of being optimal. These results were used for interpretation.

## Results

### Literature search

**Figure 1:**
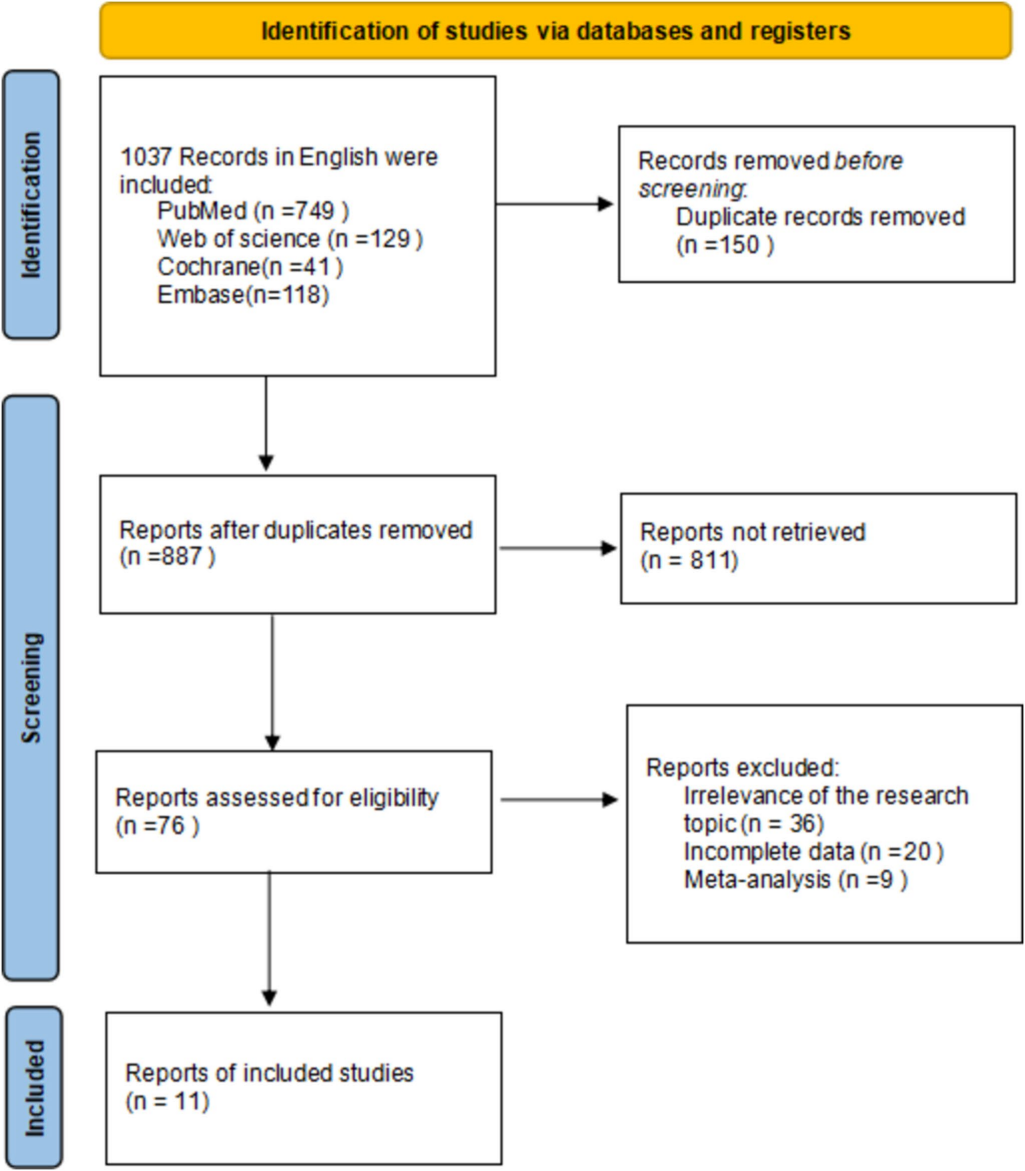
Flow diagram of the literature search.

In this study, a literature search was conducted according to inclusion and exclusion criteria, resulting in a total of 1,037 English-language documents being identified. The databases searched included PubMed (*n* = 759), Web of Science (*n* = 129), Cochrane (*n* = 41), and Embase (*n* = 118). After screening the titles and abstracts, 11 documents were selected for inclusion in the study (14, 15, 19–27), all of which were RCTs. The differences between the data of the two groups were not statistically significant (*p* > 0.05) and were comparable. The search process is illustrated in Figure 1.

### Study characteristics

**Figure 2:**
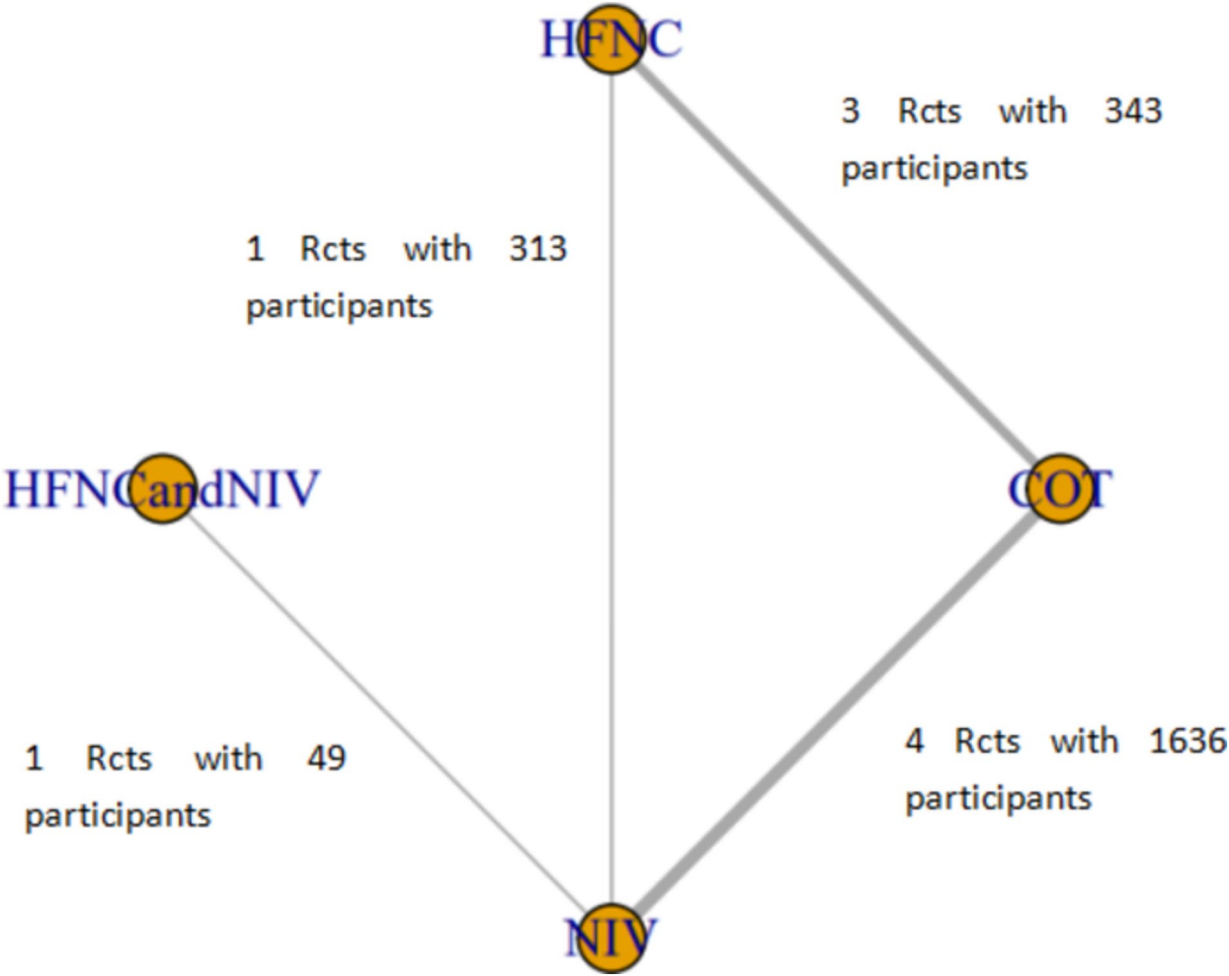
Network of the comparisons of the incidence of post-intubation SpO_2_ <80% in the Bayesian network meta-analysis.

**Figure 3:**
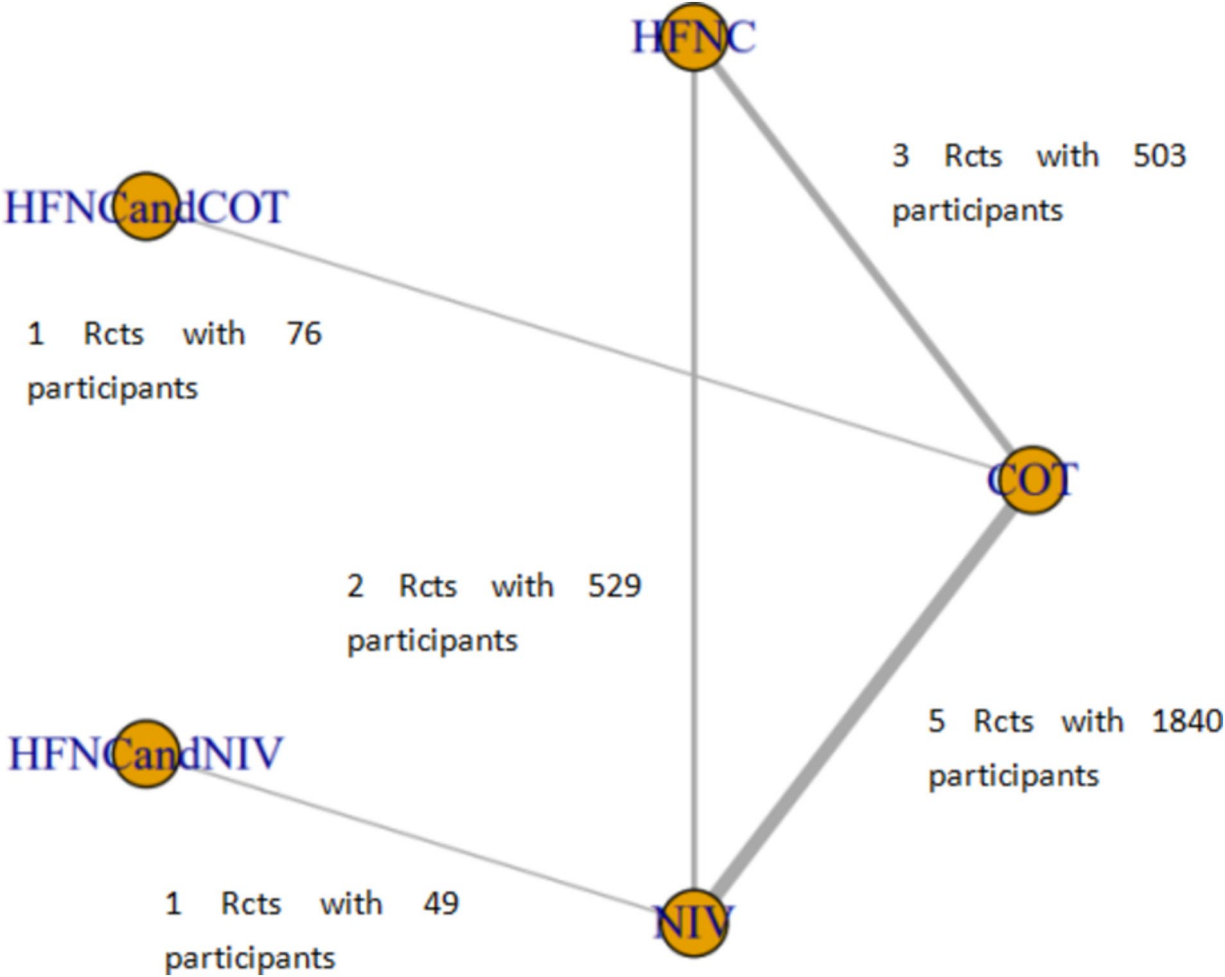
Network of the comparisons of post-intubation-related complications in the Bayesian network meta-analysis.

Six studies compared HFNC with COT, five studies compared NIV with COT, and two studies compared HFNC with NIV. Additionally, one study compared COT with a combination of HFNC and COT, and another study compared NIV with a combination of HFNC and NIV. The basic characteristics of the included literature are shown in Table 1. The grading of the evidence for each study’s outcome metrics is shown in Table 2. The network geometries are illustrated in Figures 2, 3.

**Table 1:** The basic characteristics of the included literature.

			Quality assessment					
Study, year	Country	Study design	Total No. of patients	Mean age (years)		Intervention		Outcomes
						Main exposure	Comparator	
Shahul Hameed, 2024	India	RCT	76	48 ± 2.8	44 ± 2.5	HFNC and BVM	BVM	Lowest SpO_2_ during intubation, post-intubation-related complications, ICU mortality
Simon, 2016	Germany	RCT	40	54 ± 13	63 ± 10	HFNC 50 L/min, FiO_2_: 1.0	10 L/min	The incidence of post-intubation SpO_2_ <80%, post-intubation-related complications
Vourc’h, 2015	France	RCT	119	64.9 ± 14	59.3 ± 14.5	HFNC 60 L/min, FiO_2_: 1.0	15 L/min	The incidence of post-intubation SpO_2_ <80%, lowest SpO_2_ during intubation, post-intubation-related complications, ICU LOS, ICU mortality
Guitton, 2019	France	RCT	184	60.35 ± 17.31	56.42 ± 20.35	HFNC 60 L/min, FiO_2_: 1.0	15 L/min	The incidence of post-intubation SpO_2_ <80%, lowest SpO_2_ during intubation, post-intubation-related complications, ICU LOS, ICU mortality
Baillard, 2006	France	RCT	53	64 ± 11	60 ± 15	NIV tidal volume 7–10 mL/kg, FiO_2_: 1.0, PEEP: 5 cmH_2_O	15 L/min	The incidence of post-intubation SpO_2_ <80%, post-intubation-related complications, ICU LOS, ICU mortality
Baillard, 2018	France	RCT	201	65.35 ± 12.79	63.06 ± 15.79	NIV tidal volume 6–8 mL/kg, FiO_2_: 1.0, PEEP: 5 cmH_2_O	15 L/min	The incidence of post-intubation SpO_2_ <80%, lowest SpO_2_ during intubation, post-intubation-related complications, ICU LOS, ICU mortality
Frat, 2019	France	RCT	313	64 ± 13	64 ± 14	NIV tidal volume 6–8 mL/kg, FiO_2_: 1.0, PEEP: 5 cmH_2_O	HFNC 60 L/min, FiO_2_: 1.0	The incidence of post-intubation SpO_2_ <80%, lowest SpO_2_ during intubation post-intubation-related complications, ICU LOS, ICU mortality
Jaber, 2016	France	RCT	49	62.64 ± 8.65	60.86 ± 9.46	HFNC 60 L/min, FiO_2_: 1.0 and NIV FiO_2_: 1.0, PEEP: 5 cmH_2_O	NIV FiO_2_:1.0, PEEP: 5 cmH2O	The incidence of post-intubation SpO_2_ <80%, lowest SpO_2_ during intubation, post-intubation-related complications, ICU LOS, ICU mortality
Frat, 2015	France	RCT	310	61 ± 16	59 ± 17 61 ± 17	HFNC 50 L/min, FiO_2_: 1.0	10 L/min, NIV tidal volume 7–10 mL/kg PEEP: 2–10 cmH_2_O	Post-intubation related complications, ICU mortality
Brambilla, 2014	Italy	RCT	81	64.9 ± 16.1	69.5 ± 15.8	NIV 90–140 L/min, PEEP: 10 cmH_2_O	COT	The incidence of post-intubation SpO_2_ <80%, post-intubation-related complications, ICU LOS, ICU mortality
Gibbs, 2024	USA	RCT	1,301	60.9 ± 3.9	60.9 ± 3.7	NIV FiO_2_: 100%,	COT	The incidence of post-intubation SpO_2_ <80%, lowest SpO_2_ during intubation, post-intubation-related complications, ICU mortality

HFNC, high-flow nasal cannula; NIV, non-invasive ventilation; COT, conventional oxygen therapy; PEEP, positive end-expiratory pressure.

**Table 2:** The grading of the evidence for each study’s outcome metrics.

	Certainty assessment							No. of patients		Certainty
	No. of studies	Study design	Risk of bias	Inconsistency	Indirectness	Imprecision	Other considerations	Experimental group	control group	
①	9	RCT	Not serious	Serious^a^	Not serious	Serious^b^	None	104/1,154	182/1,187	⊕⊕⊙⊙
②	7	RCT	Not serious	Not serious	Not serious	Serious^b^	None	1,106	1,137	⊕⊕⊕⊙
③	10	RCT	Not serious	Serious^a^	Not serious	Serious^b^	None	450/1,388	453/1,299	⊕⊕⊙⊙
④	7	RCT	Not serious	Serious^a^	Not serious	Serious^b^	None	490	510	⊕⊕⊙⊙
⑤	10	RCT	Not serious	Serious^a^	Not serious	Serious^b^	None	399/1,389	402/1,298	⊕⊕⊙⊙

① The incidence of post-intubation SpO_2_ <80%, ② lowest SpO_2_ during intubation, ③ post-intubation-related complications, ④ ICU length of stay, and ⑤ ICU mortality. ⊕⊕⊙⊙ Low. ⊕⊕⊕⊙ Medium.

a Down one level: inconsistent outcome indicators.

b Down one level: sample size of study too small.

### Risk of bias

**Figure 4:**
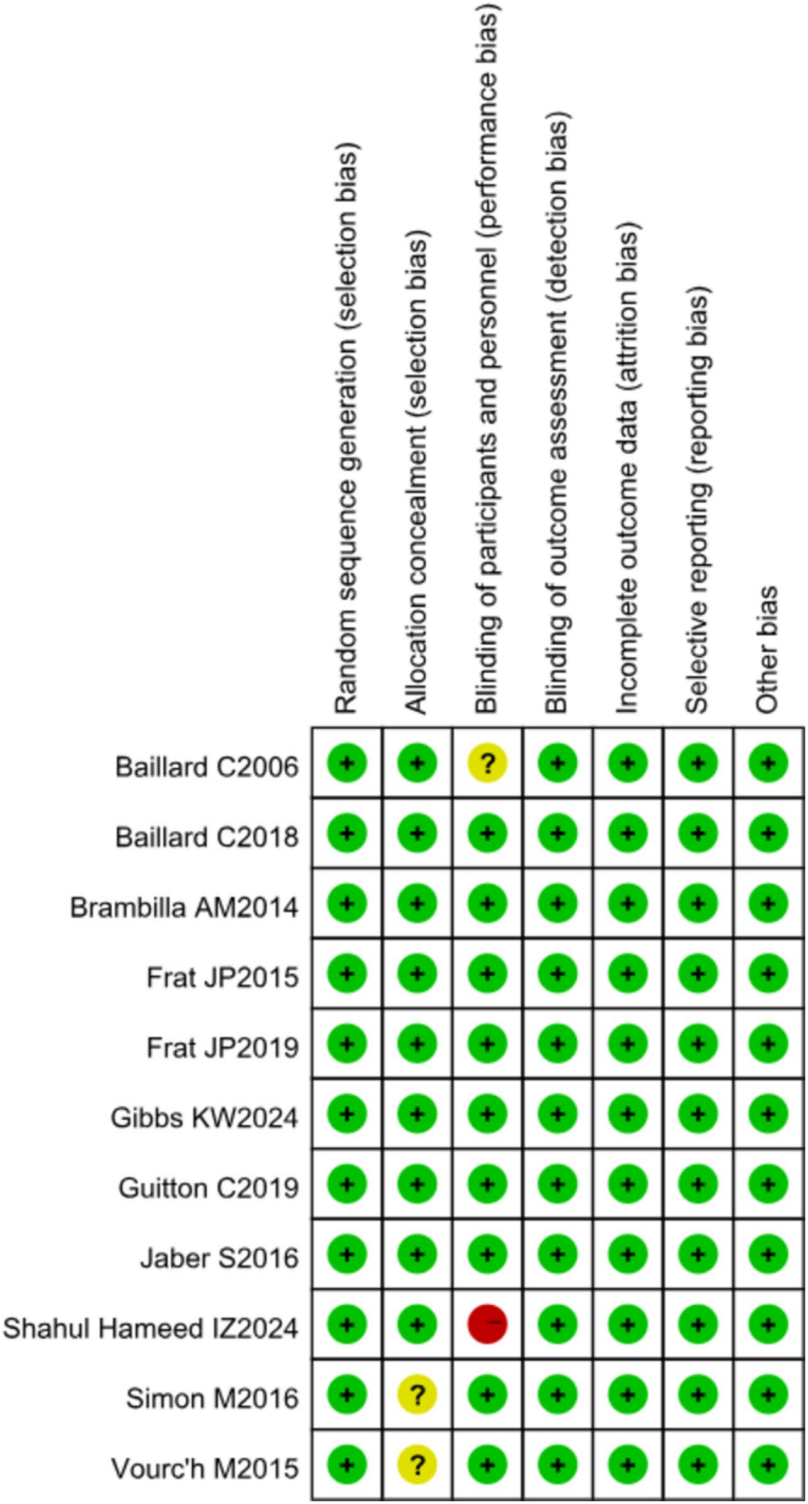
Risk of bias of included studies.

One study had a high risk of bias, four studies had an unclear risk of bias, and five studies had a low risk of bias. The overall risk of bias for the included literature is shown in Figure 4.

### The incidence of post-intubation SpO_2_ <80%

**Figure 5:**
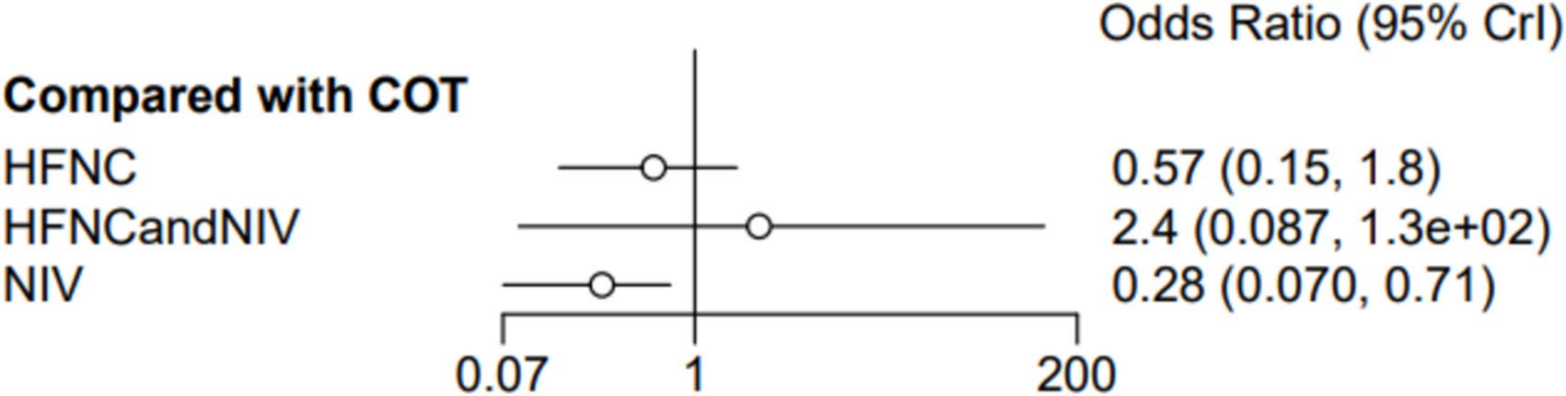
Forest plot of the incidence of post-intubation SpO_2_ <80%. HFNC, high-flow nasal cannula; NIV, non-invasive ventilation; COT, conventional oxygen therapy.

**Figure 6:**
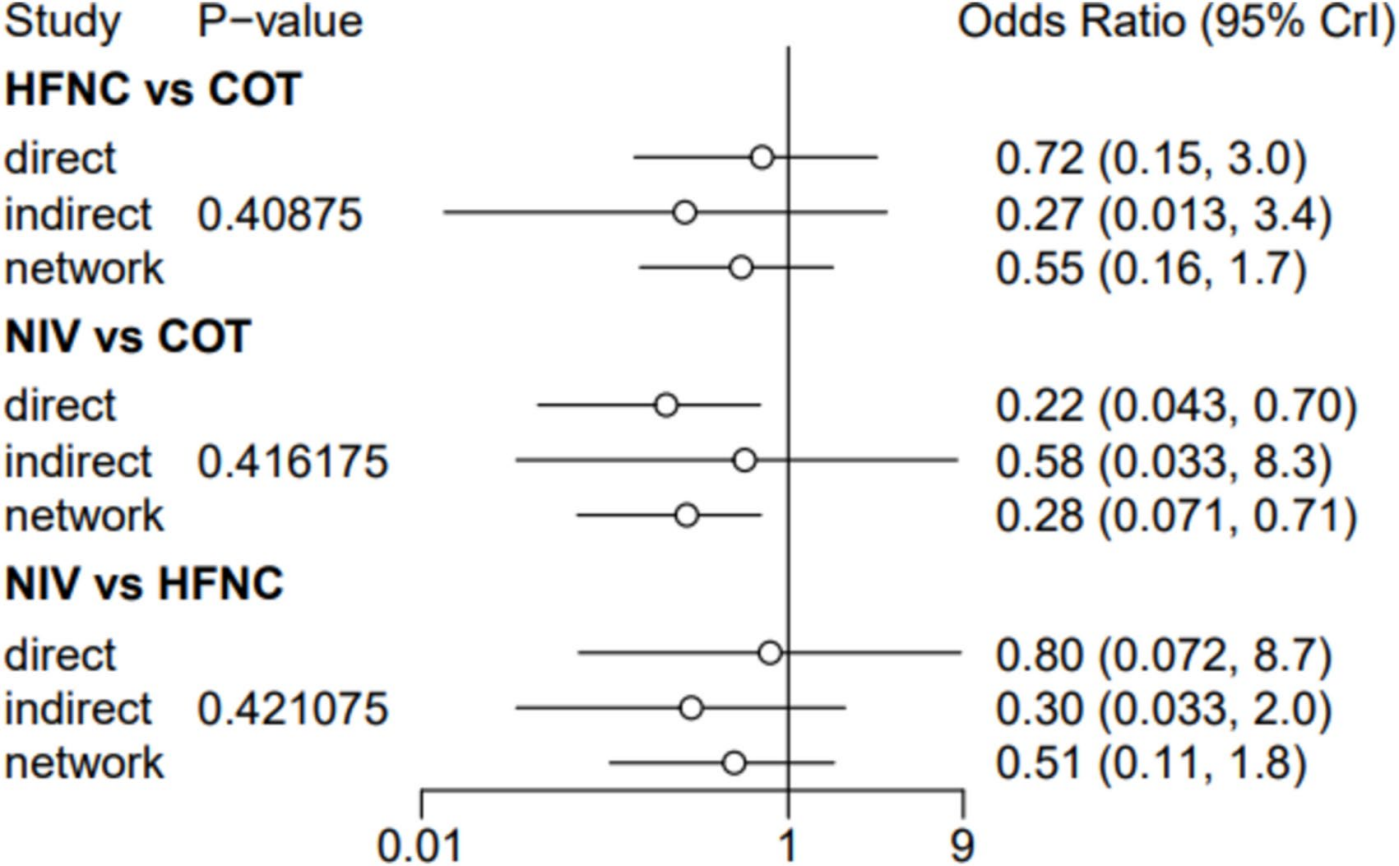
A node-split analysis of the incidence of post-intubation SpO_2_ <80%.

**Figure 7:**
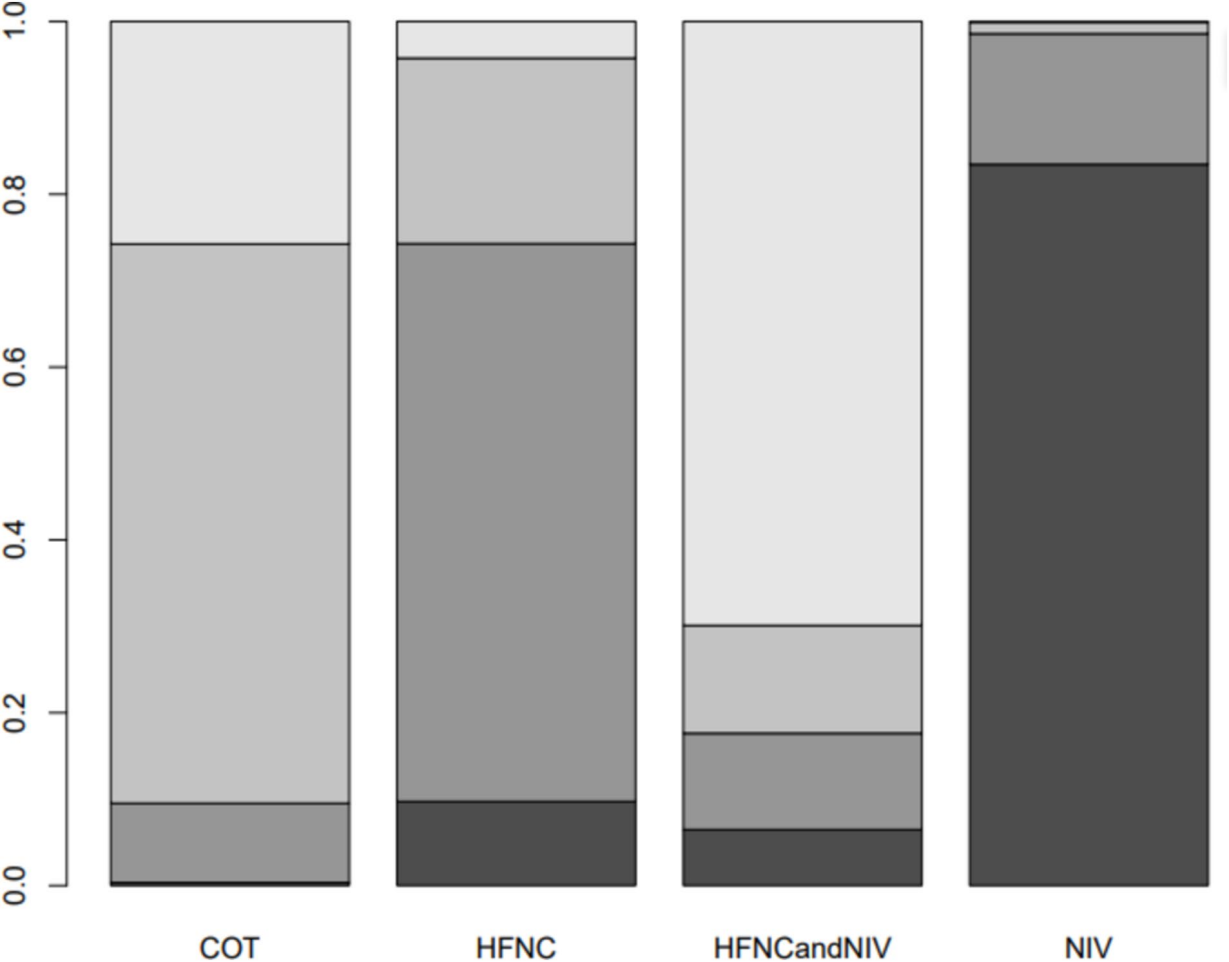
Bar chart showing the surface under the cumulative ranking curve (SUCRA) values for the incidence of post-intubation SpO_2_ <80%.

Nine trials (2,341 patients) reported the incidence of SpO_2_ <80% in intubation. The network geometry is shown in Figure 2. Direct comparisons showed that compared to COT preoxygenation, NIV preoxygenation (RR 0.28, 95% CI 0.070, 0.71), HFNC combined with NIV (RR 2.4, 95% CI 0.087, 1.3), and HFNC (RR 0.28, 95% CI 0.070, 0.71), NIV preoxygenation in intubation had a significant SpO_2_ <80% difference (Figure 5). We also performed a node-split analysis to assess the inconsistency in the network meta-analysis and found no significant differences between COT vs. HFNC (*p* = 0.409), COT vs. NIV (*p* = 0.416), and HFNC vs. NIV (*p* = 0.421), suggesting a high degree of consistency in the results of the direct and indirect comparisons between the three groups (Figure 6). In the rank-ordering plot, the NIV preoxygenation group (83.46%) was the highest, while the COT preoxygenation group (0.34%) was the lowest (Figure 7). The results of the rank-ordering plot showed that the NIV group (83.46%) was superior to the HFNC group (9.73%), the HFNC combined with the NIV group (6.48%), and the COT group (0.34%) in terms of the lowest SpO_2_ during intubation (Supplementary Table S1). The level of evidence was low (Table 2).

### Lowest SpO_2_ during intubation

**Figure 8:**
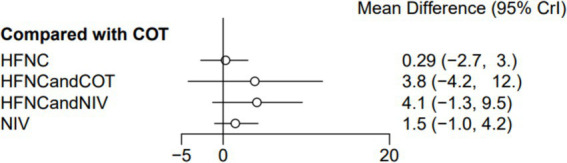
Forest plot of the lowest SpO_2_ during intubation. HFNC, high-flow nasal cannula; NIV, non-invasive ventilation; COT, conventional oxygen therapy.

**Figure 9:**
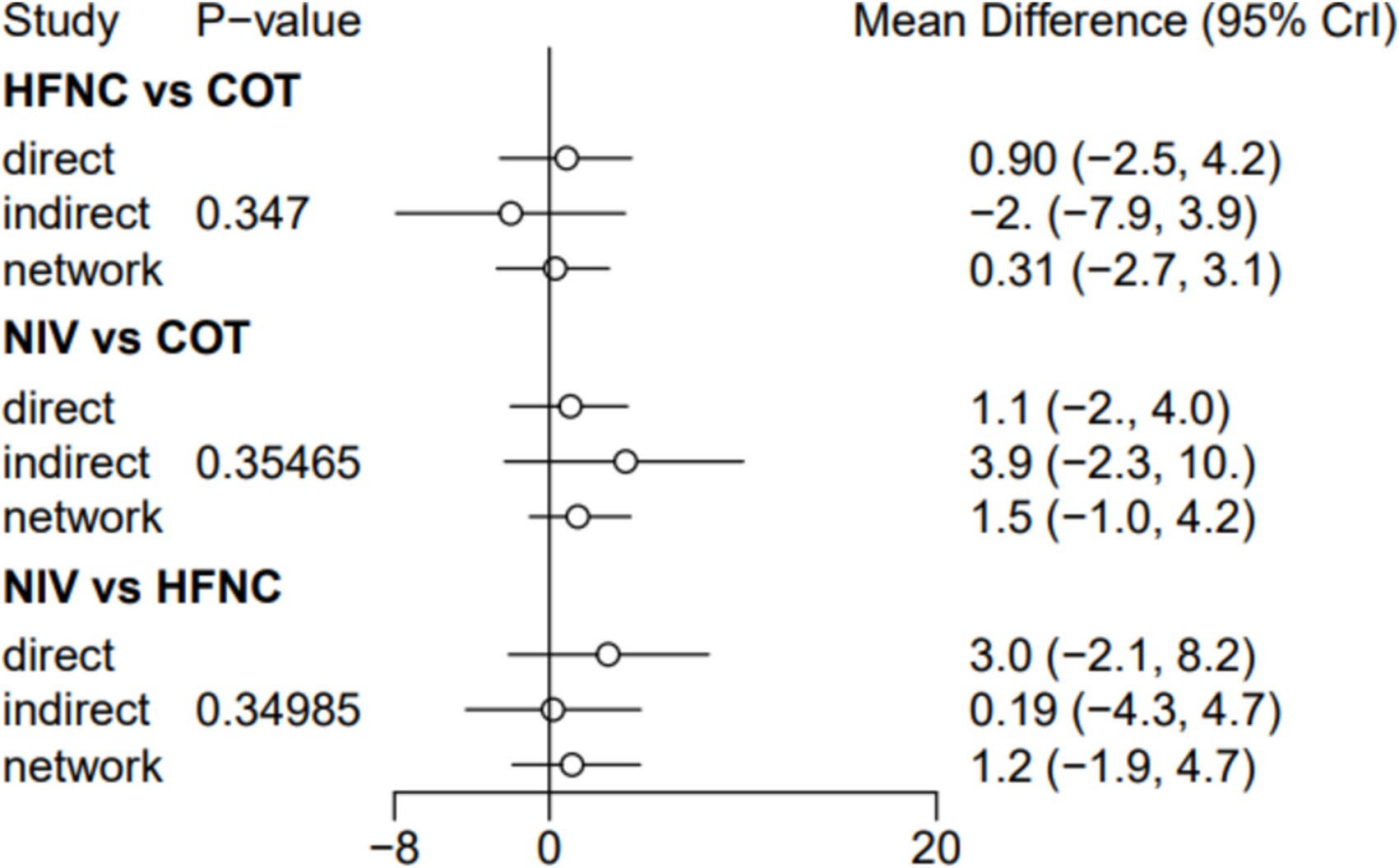
A node-split analysis of the lowest SpO_2_ during intubation.

**Figure 10:**
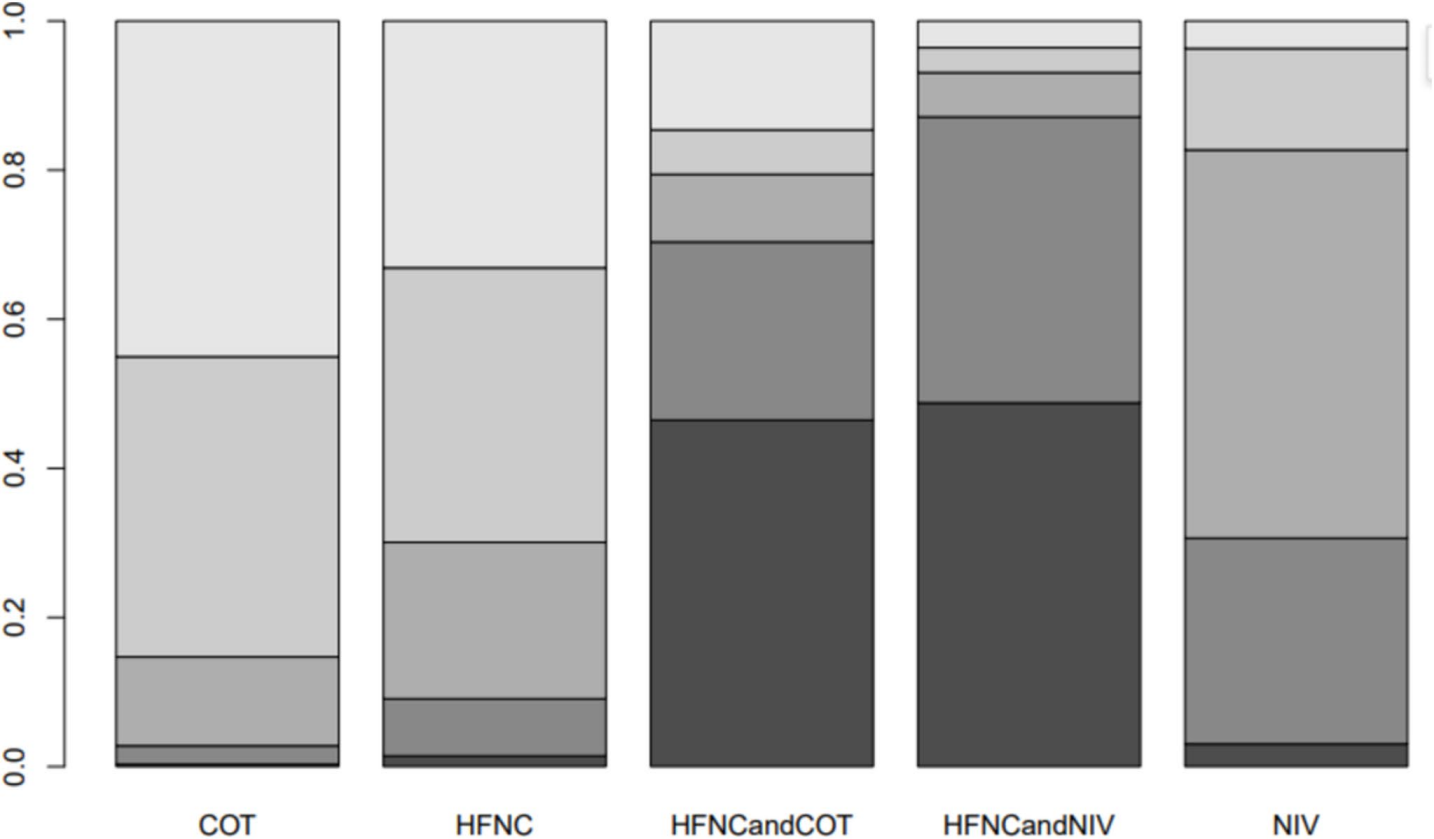
Bar chart showing the surface under the cumulative ranking curve (SUCRA) values for the lowest SpO_2_ during intubation.

Seven trials (2,243 patients) reported the lowest SpO_2_ during intubation. The network geometry is shown in Supplementary Figure S6. Direct comparisons showed that, compared to COT preoxygenation, HFNC preoxygenation (MD 0.29, 95% CI −2.7, 3.0), HFNC combined with COT (MD 3.8, 95% CI −4.2, 12.0), HFNC combined with NIV (MD 4.1, 95% CI −1.3, 9.5), and NIV preoxygenation (MD 1.5, 95% CI −1.0, 4.2) all showed no significant difference in the lowest SpO_2_ during intubation (Figure 8). We also performed a node-split analysis to assess the inconsistency in the network meta-analysis and found no significant differences between COT vs. HFNC (*p* = 0.35), COT vs. NIV (*p* = 0.35), and HFNC vs. NIV (*p* = 0.35), suggesting a high degree of consistency in the results of the direct and indirect comparisons between the three groups (Figure 9). In the rank-ordering plot, the HFNC combined with the COT preoxygenation group (49.98%) was the highest, while the COT preoxygenation group (0.70%) was the lowest (Figure 10). The results of the rank-ordering plot showed that the HFNC combined with the NIV group (48.78%) was superior to the HFNC combined with the COT group (46.47%), the NIV group (3.04%), the HFNC group (1.42%), and the COT group (0.30%) in terms of the lowest SpO_2_ during the intubation (Supplementary Table S1). The level of evidence was moderate (Table 2).

### Post-intubation-related complications

**Figure 11:**
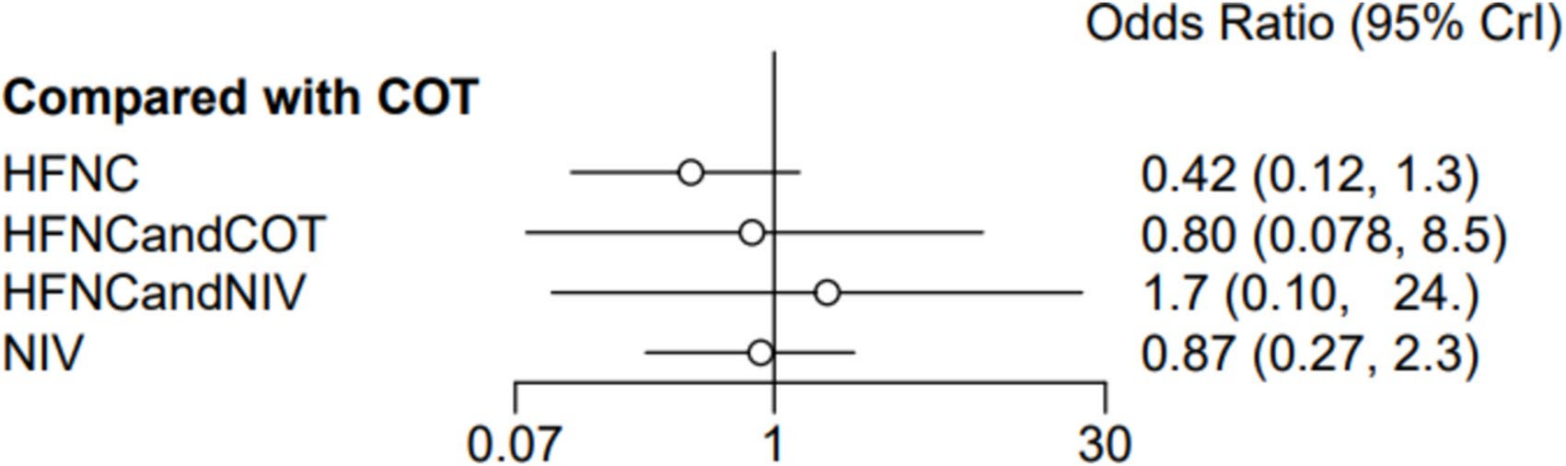
Forest plot of post-intubation-related complications. HFNC, high-flow nasal cannula; NIV, non-invasive ventilation; COT, conventional oxygen therapy.

**Figure 12:**
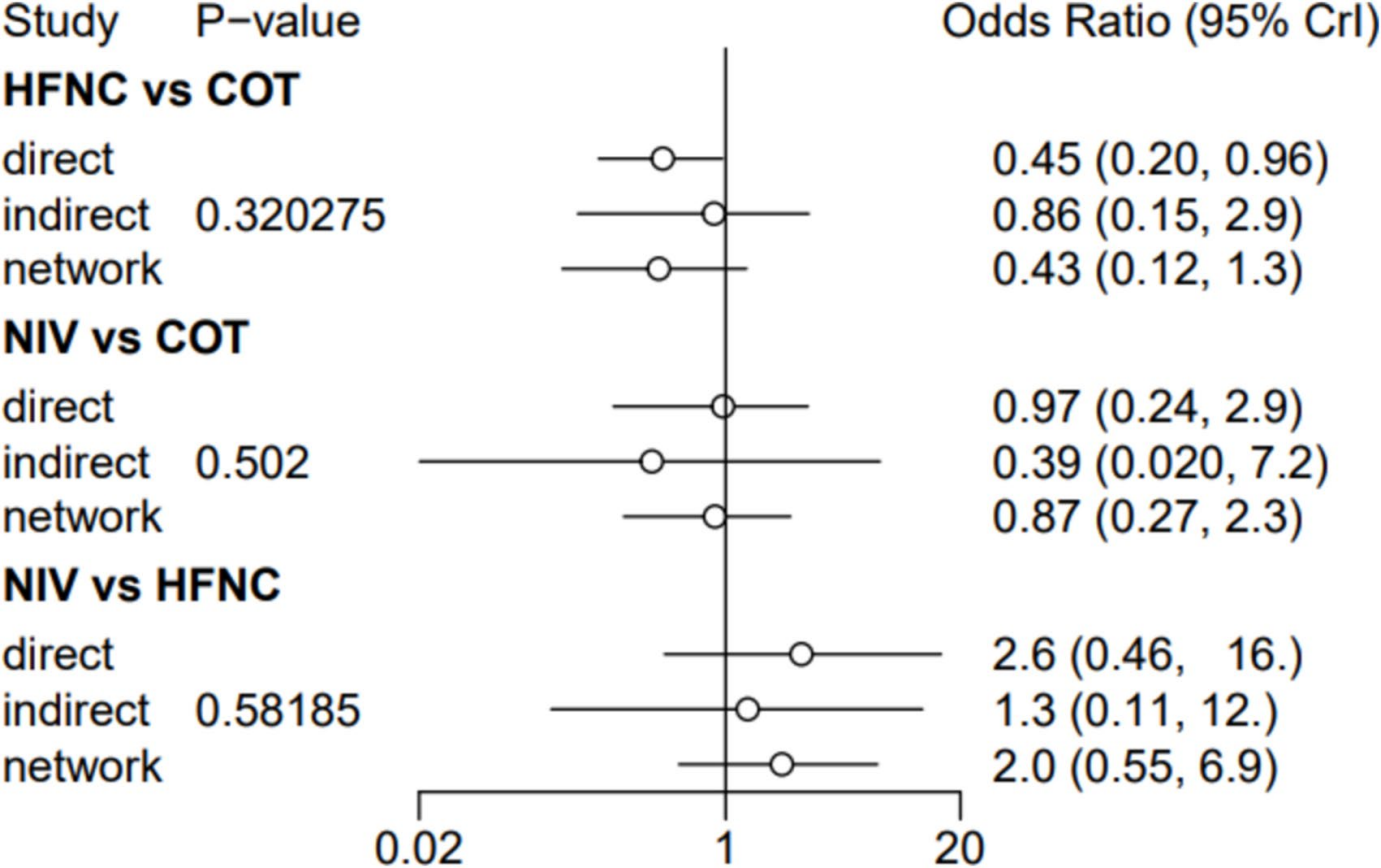
A node-split analysis of post-intubation-related complications.

**Figure 13:**
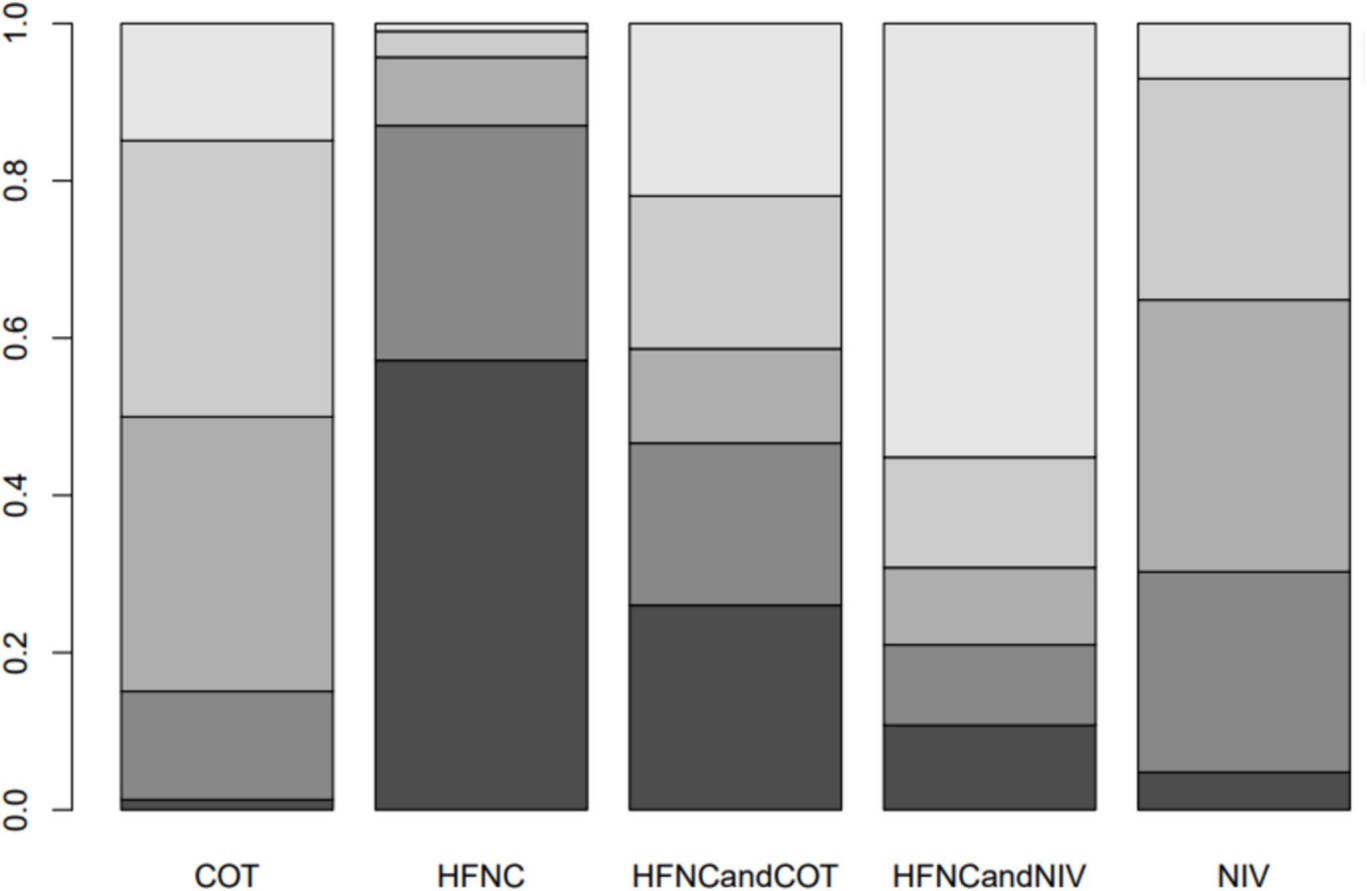
Bar chart showing the surface under the cumulative ranking curve (SUCRA) values for post-intubation-related complications.

Ten trials (2,687 patients) reported intubation-related complications. Direct comparisons showed no significant differences in any of the other preoxygenation measures compared to COT preoxygenation (see Figure 11). We also performed a node-split analysis to assess the inconsistency in the network meta-analysis and found no significant differences between COT vs. HFNC (*p* = 0.32), COT vs. NIV (*p* = 0.50), and HFNC vs. NIV (*p* = 0.58), suggesting that the results of the direct and indirect comparisons between the three groups were highly consistent (Figure 12). In the rank-ordering plot, the HFNC preoxygenation group (57.16%) was the highest, while the COT group (1.28%) was the lowest (Figure 13). The results of the rank-ordering plot showed that the HFNC group (57.16%) was superior to the HFNC combined with the COT group (26.02%), the HFNC combined with the NIV group (10.76%), the NIV group (4.78%), and the COT group (1.28%) in terms of the lowest SpO_2_ during intubation (Supplementary Table S1). The level of evidence was low (Table 2).

### ICU length of stay

**Figure 14:**
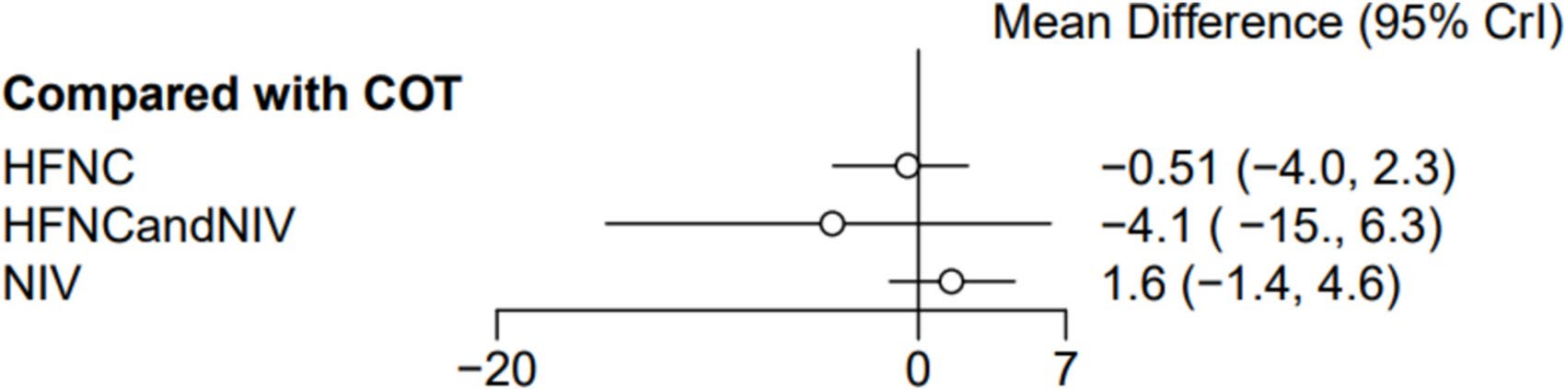
Forest plot of ICU LOS. HFNC, high-flow nasal cannula; NIV, non-invasive ventilation; COT, conventional oxygen therapy.

Six trials (1,000 patients) reported the ICU length of stay (LOS). Direct comparisons showed no significant differences in any of the other preoxygenation measures compared to COT preoxygenation (see Figure 14). Node-split analyses were performed to assess the inconsistency in the network meta-analyses, revealing no significant differences between COT vs. HFNC (*p* = 0.39), COT vs. NIV (*p* = 0.40), and HFNC vs. NIV (*p* = 0.40). This suggests that the results of direct and indirect comparisons among the three groups were highly consistent (Supplementary Figure S9). In the rank-ordering plot, the HFNC preoxygenation group (59.41%) ranked the highest, while the NIV preoxygenation group (2.80%) ranked the lowest (Supplementary Figure S10). The results indicated that the HFNC group (59.41%) was superior to the COT group (30.75%), the HFNC combined with the NIV group (7.04%), and the NIV group (2.80%) in terms of the lowest SpO_2_ during intubation (Supplementary Table S1). However, the level of evidence was low (Table 2).

### ICU mortality

**Figure 15:**
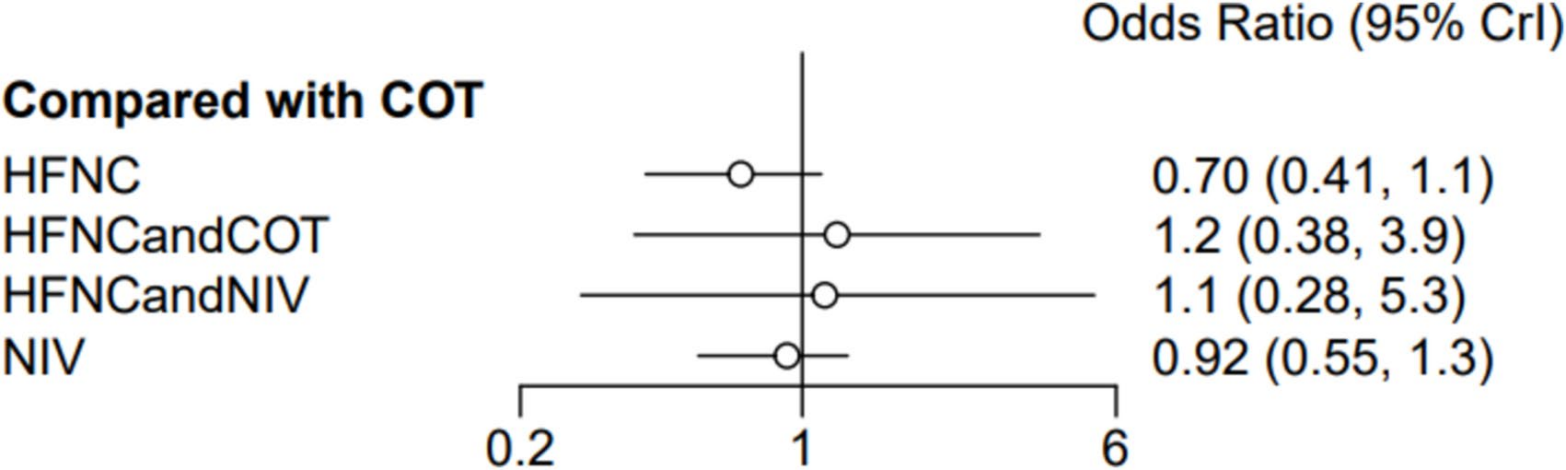
Forest plot of ICU mortality. HFNC, high-flow nasal cannula; NIV, non-invasive ventilation; COT, conventional oxygen therapy.

Ten trials (2,687 patients) reported ICU mortality. Direct comparisons showed no significant differences in any of the other preoxygenation measures compared to COT preoxygenation (see Figure 15). We also performed node-split analyses to assess the inconsistency in the network meta-analyses and found no significant differences between COT vs. HFNC (*p* = 0.89), COT vs. NIV (*p* = 0.80), and HFNC vs. NIV (*p* = 0.30), suggesting a high degree of concordance of the results of direct and indirect comparisons between the three groups (Supplementary Figure S10). In the rank-ordering plot, the HFNC preoxygenation group (57.29%) ranked the highest, while the COT preoxygenation group (1.41%) ranked the lowest (Supplementary Figure S11). The results of the rank-ordering plot showed that the HFNC group (57.29%) was superior to the HFNC combined with the NIV group (21.07%), the HFNC combined with the COT group (13.90%), the NIV group (6.34%), and the COT group (1.41%) in terms of the lowest SpO_2_ during intubation (Supplementary Table S1). The level of evidence was low (Table 2).

### Publication bias

**Figure 16:**
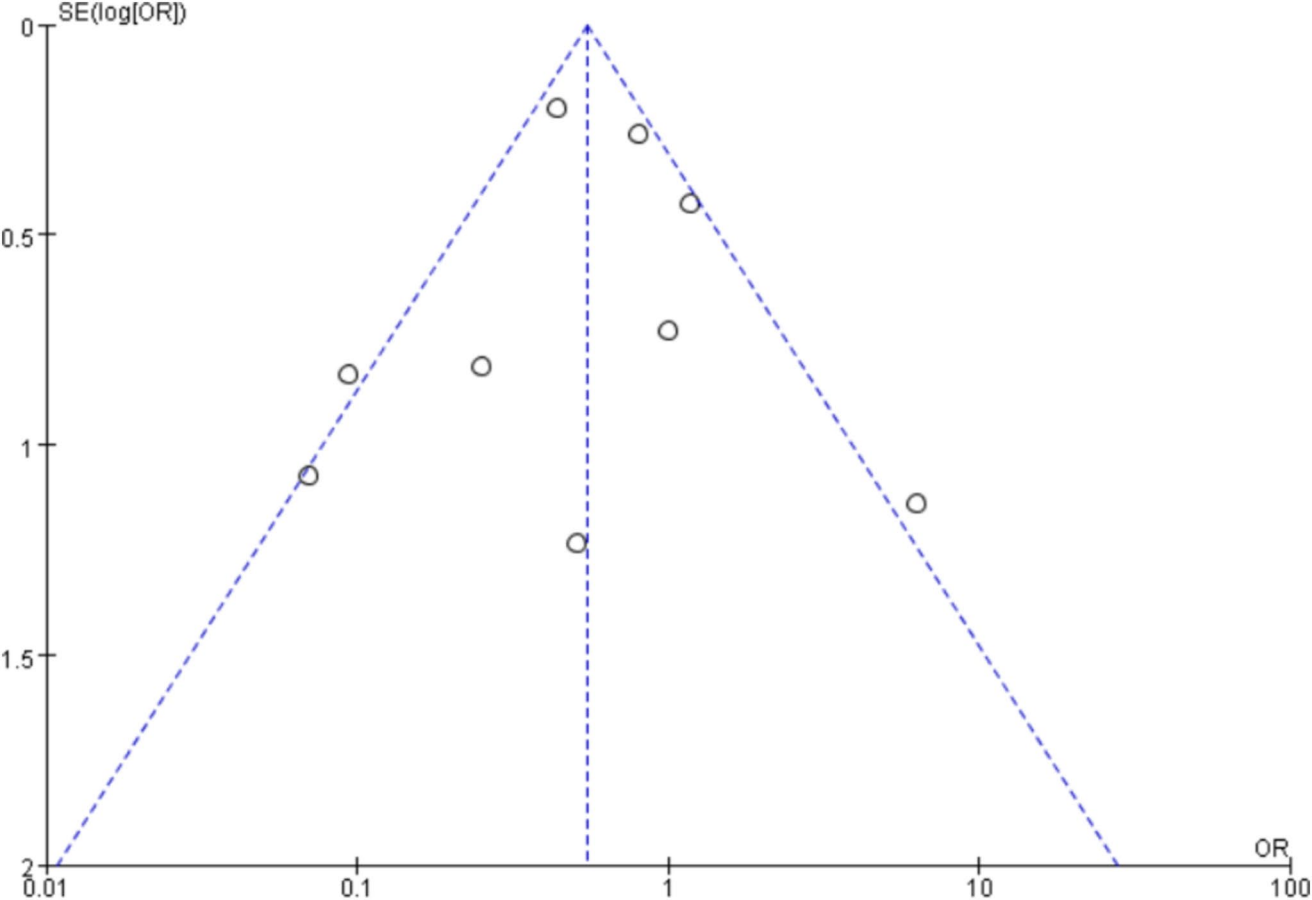
Funnel plot as an indicator of the incidence of SpO_2_ <80% after intubation.

A funnel plot was used to plot the incidence of post-intubation SpO_2_ <80% analyzed in this article, and no significant publication bias was seen, as shown in Figure 16, where each point is within the range of the funnel plot, and the overall image is essentially symmetrical, suggesting that there is a relatively low likelihood of significant publication bias.

## Discussion

While preoxygenation improves ETI in AHRF patients, optimal strategies remain unclear. This network meta-analysis of 11 RCTs (1,573 patients) compared five preoxygenation methods: COT, HFNC, NIV, HFNC combined with COT, and HFNC combined with NIV. NIV significantly reduced SpO_2_ <80% incidence compared to other methods; HFNC combined with COT and HFNC combined with NIV minimized SpO_2_ decreases during intubation. HFNC was associated with lower risks of intubation-related complications (new infiltrates, hemodynamic instability, cardiac arrest), reduced ICU length of stay, and lower mortality. HFNC may thus provide superior oxygenation during ETI in AHRF, improving patient outcomes. In AHRF, lung inflammation and pulmonary edema cause hypoxemia. Ventilatory impairment increases respiratory burden. Preoxygenation improves oxygenation during ETI. Although higher PaCO_2_ levels were observed with HFNC preoxygenation during anesthesia induction in head and neck surgery (28), preoxygenation significantly reduced hypoxemia in emergency surgery (29–31) and, in obese patients, increased safe apnea time while maintaining SpO_2_ in high-risk individuals (32). Obese patients exhibit altered respiratory physiology characterized by reduced functional residual capacity (FRC), lower tidal volumes, decreased compliance, increased oxygen demand, and a higher risk of atelectasis. These factors contribute to significantly faster desaturation post-induction compared to non-obese individuals. Preoxygenation is critical during emergency ETI, as it enhances oxygen reserves, improves ventilation and oxygenation, reduces respiratory burden, relieves dyspnea, shortens the length of stay in the ICU, and improves survival during emergency ETI, except in patients who are on muscle relaxants or who are not spontaneously breathing. Further multicenter studies are needed to evaluate HFNC preoxygenation in AHRF patients during intubation.

### Incidence of post-intubation SpO_2_ <80%

The meta-analysis of nine RCTs demonstrated that NIV preoxygenation significantly reduced post-intubation SpO_2_ <80%. Baillard et al. (22) reported significantly fewer instances of SpO_2_ <80% in AHRF patients preoxygenated with NIV (expiratory tidal volume 7–10 mL/kg, FiO_2_ 100%, PEEP 5 cmH_2_O) compared to COT. A US multicenter study similarly showed a lower incidence of SpO_2_ <85% with NIV, representing an ~9.4% reduction in hypoxemia. NIV improves oxygenation via high oxygen concentration delivery, reduces respiratory muscle burden, and increases lung volume. Significant improvements in oxygenation were observed after 3 min of use.

Frat et al. (24) defined severe hypoxemia as a sustained SpO_2_ <80% for ≥5 s post-preoxygenation. Although not statistically significant, severe hypoxemia occurred in 23% of patients following NIV preoxygenation and 27% following HFNC preoxygenation. However, a subgroup analysis of patients with moderate-to-severe hypoxemia (PaO_2_/FiO_2_ ≤200) revealed the superiority of NIV (24% vs. 35% severe hypoxemia). Another study confirmed the greater effectiveness of NIV than COT in preventing severe hypoxemia during preoxygenation in patients previously treated with NIV (23). In contrast, a comparison of obese and non-obese patients revealed a higher incidence of severe hypoxemia (SpO_2_ <80%) in the obese group; NIV preoxygenation did not reduce this risk compared to HFNC preoxygenation (33). Vourc’h et al. (15) reported three more cases of SpO_2_ <80% in the HFNC group (60 L/min, FiO_2_ 100%) compared to the COT group (15 L/min mask). However, the actual inhaled oxygen may have differed less than expected. COT’s mask delivery may provide comparable oxygenation to HFNC. Jaber et al. (14) reported only one case of SpO_2_ <80% with HFNC combined with NIV preoxygenation vs. five with NIV alone, although this difference was not significant due to the small sample size and potential overestimation of treatment effects in small studies. This is only the result of previous research, as there may be heterogeneity in the included studies, as patients receiving NIV may have varying degrees of disease severity. Therefore, more high-quality research is still needed in the future.

### Lowest SpO_2_ during intubation

This study included seven RCTs to examine the effects of various preoxygenation strategies on the lowest SpO_2_ during intubation, finding no statistically significant differences among them. In the study by Shahul Hameed et al. (19), the SpO_2_ levels were slightly higher in the HFNC combined with the COT group at 0 and 5 min post-intubation, but these differences were not statistically significant, consistent with our findings. Semler et al. (34) randomized 150 ICU patients undergoing ETI to receive preoxygenation with 100% oxygen via HFNC at >15 L/min or no supplemental oxygen during laryngoscopy, reporting mid-minimum SpO_2_ levels of 92%, with HFNC compared to 90% with COT at <15 L/min, indicating that HFNC preoxygenation alone can be effective. Jaber et al. (14) found that in critically ill patients with severe hypoxic respiratory failure, preoxygenation with HFNC combined with NIV significantly increased minimum SpO_2_ during intubation compared to NIV alone. These findings suggest that high-flow preoxygenation before intubation may be beneficial for patients with acute hypoxic respiratory failure, particularly in critical moments around intubation, and that combining it with other ventilation methods can further enhance oxygenation and patient outcomes.

### Post-intubation-related complications

The 10 included studies did not show a statistically significant difference between the various preoxygenation strategies in terms of the incidence of post-intubation complications. In the Frat et al. (24) study, comparing HFNC and NIV as preoxygenation strategies, the NIV group exhibited three cases of persistent arrhythmia, two cases of bradycardia, one case of cardiac arrest during and after intubation, and 28 cases of new infiltrates on the chest X-ray. The HFNC group had three cases of persistent arrhythmia, three cases of bradycardia during and after intubation, five cases of cardiac arrest, and 33 cases of new infiltrates on the chest X-ray. However, there was no statistical difference between the two groups in terms of various complications, consistent with the results of the present study. In the study by Guitton et al. (21), serious complications including SpO_2_ <80%, severe hypotension, and cardiac arrest occurred in 6% of patients in the HFNC group compared to 16% of patients in the COT group, indicating that HFNC significantly reduced the risk of associated complications (35). HFNC also offers greater flexibility during ETI, providing continuous oxygen delivery and facilitating easier operating conditions for medical staff while safeguarding the patient’s oxygenation needs. Although complications can still occur with various preoxygenation strategies, the differences in their effectiveness are small. Although HFNC and NIV both have different problems after long-term use, HFNC and NIV are safer and more effective than COT.

### ICU length of stay

Seven studies were included, and none of the various preoxygenation strategies were statistically significant in terms of ICU LOS. One of the included studies by Vourc’h et al. (15) showed that ICU LOS was approximately 3 to 4 days shorter in the HFNC group compared to the COT group. This suggests a potential advantage of HFNC preoxygenation in the management of patients with AHRF, although the difference was not statistically significant. The shortened LOS had a positive impact on both patient recovery and healthcare resource utilization. The results of a previous meta-analysis also showed no effect of HFNC on ICU LOS compared to NIV (36), consistent with the findings of the present study. However, in the study by Jaber et al. (14), the intervention group was treated with HFNC combined with NIV (PEEP 5 cmH_2_O, FiO_2_ 100%) at an oxygen flow rate of 60 L/min, while the control group received NIV only (with the same parameters as in the intervention group), both preoxygenated for 4 min. The LOS in the intervention group was prolonged by almost a week compared to the control group, although there was no statistically significant difference between the two groups. The prolonged LOS in the intervention group, contrary to expectations, may involve various factors such as individual patient differences, disease severity, complication rates, treatment adherence, and sample size. Only 49 patients were included in this study, making the results underrepresentative due to the limited sample size. Future studies need to consider expanding the sample size to improve the stability and reliability of the results and provide more effective guidance for clinical practice.

### ICU mortality

None of the various preoxygenation strategies included in the 10 studies were statistically significant in terms of ICU mortality. In Baillard’s 2006 study, ICU mortality was 30% in the NIV preoxygenation group compared to 50% in the COT preoxygenation group. Although NIV reduced ICU mortality, there was no significant difference between the two groups, consistent with the results of this study. Previous meta-analyses have reached relatively contradictory conclusions regarding the effect of NIV on mortality in patients with AHRF, with 31 of 252 patients (12.3%) treated with NIV dying in the hospital, compared to 62 of 251 patients (24.7%) who received standard oxygen therapy. Clearly, the NIV group had a significant reduction in hospital mortality (37).

In Frat’s et al. (25) study, patients were divided into three groups: the COT group was administered oxygen by mask at 10 L/min; the HFNC group received oxygen through a heated and humidified apparatus with a gas flow rate of 50 L/min, initially with a FiO_2_ of 1.0; and in the NIV group, non-invasive ventilation was provided using respiratory masks with pressure support adjusted to achieve an expiratory tidal volume of 7 to 10 mL/kg of predicted body weight and an initial positive end-expiratory pressure (PEEP) between 2 and 10 cmH_2_O. The number of ICU deaths in this study was 31 in the NIV group, 13 in the HFNC group, and 22 in the COT group, with statistically significant results. These data suggest that the effect of HFNC preoxygenation during ETI in patients with AHRF may be the most significant and outstanding compared to COT and NIV, especially in terms of ICU mortality. This also suggests that HFNC has the potential to improve the prognosis of patients.

This study has several limitations. First, the small number of included articles (only 11) and the fact that some of the outcome indicators were only mentioned in individual papers may have affected the test efficacy and led to potential differences in the results. Second, some of the articles had small sample sizes, which limited their representativeness. Third, a publication bias assessment could not be conducted due to the limited number of included articles. Fourth, it may be due to differences in expertise and institutional protocols between clinicians and nurses in multiple studies. Finally, the lack of a unified standard for the devices used in preoxygenation and the variations in the settings of values across the studies contributed to group differences and resulted in high heterogeneity in some analyses, which may hinder future clinical development.

## Conclusion

In a meta-analysis of preoxygenation strategies during ETI in patients with AHRF, the use of NIV preoxygenation appeared to be effective in reducing the incidence of SpO_2_ <80%. However, HFNC preoxygenation was found to be safer and more effective in terms of reducing the emergence of associated complications after intubation, ICU LOS, and mortality. Further studies are needed to better understand the relative benefits of each strategy.

## Data Availability

The original contributions presented in the study are included in the article/Supplementary material, further inquiries can be directed to the corresponding authors.
