# Supplementary material for: Preoxygenation strategies before intubation in patients with acute hypoxic respiratory failure: a network meta-analysis
Source: Front Med (Lausanne). 2025 Feb 10;12:1532911. doi: 10.3389/fmed.2025.1532911 (PMC11847861; doi:10.3389/fmed.2025.1532911)
Supplement: Supplementary file 1 [file Data_Sheet_1.doc]

Additional file


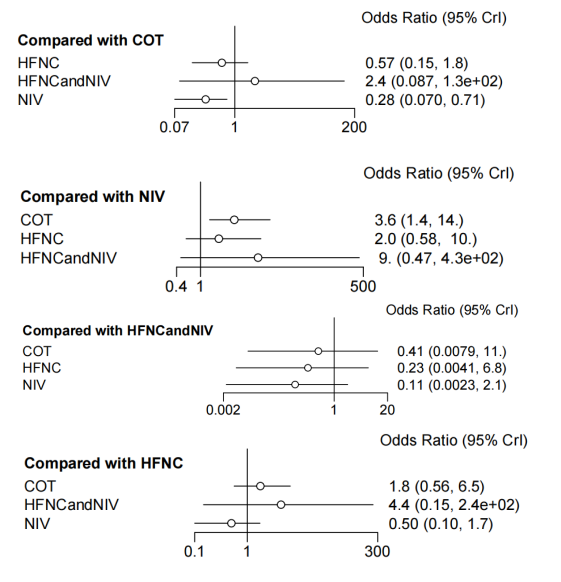


**Figure S1** Forest plot of the incidence of post-intubation SpO2 <80%. HFNC:high-flow nasal cannula ,NIV:non-invasive ventilation ,COT:conventional oxygen therapy.


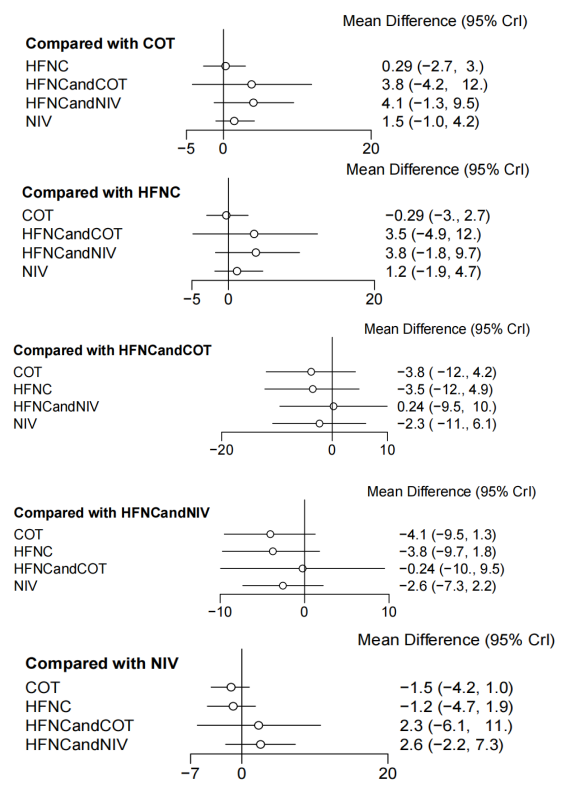


**Figure S2** Forest plot of lowest SpO2 during intubation. HFNC:high-flow nasal cannula ,NIV:non-invasive ventilation ,COT:conventional oxygen therapy.


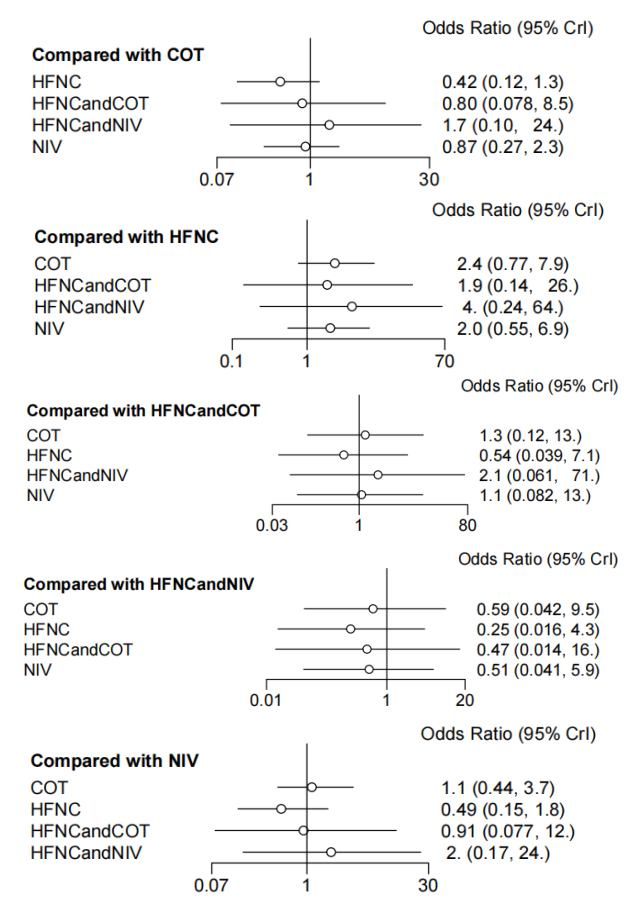


**Figure S3** Forest plot of post-intubation related complications. HFNC:high-flow nasal cannula ,NIV:non-invasive ventilation ,COT:conventional oxygen therapy.


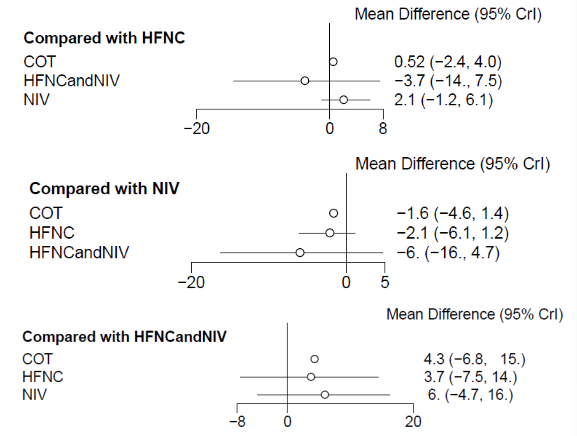


Figure S4 Forest plot of ICU length of stay. HFNC:high-flow nasal cannula ,NIV:non-invasive ventilation ,COT:conventional oxygen therapy.


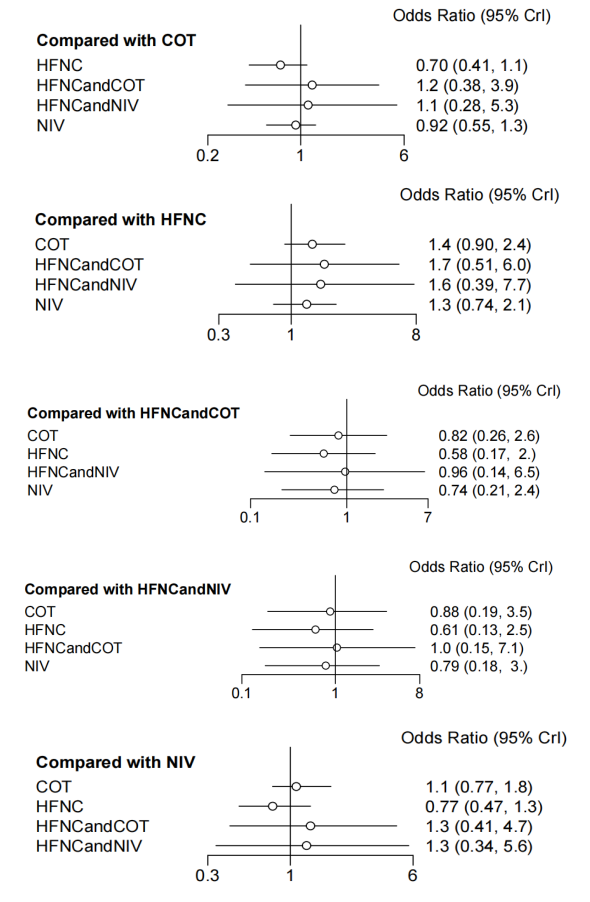


**Figure S5** Forest plot of ICU mortality. HFNC:high-flow nasal cannula ,NIV:non-invasive ventilation ,COT:conventional oxygen therapy.

**
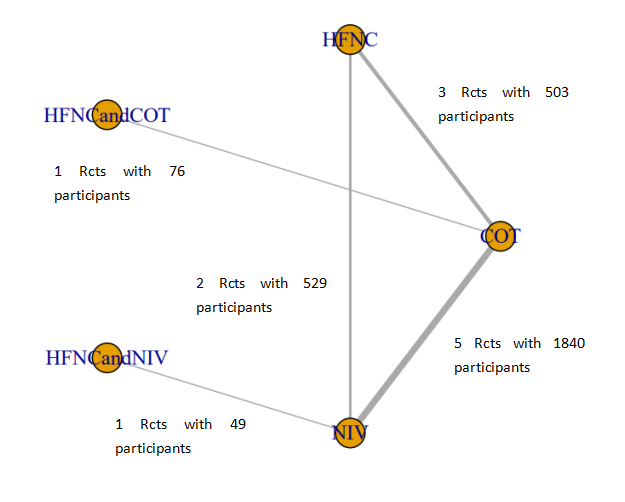
**

**Figure S6** Network of the comparisons of post-intubation related complications in the Bayesian network meta-analysis. HFNC:high-flow nasal cannula ,NIV:non-invasive ventilation ,COT:conventional oxygen therapy.

**
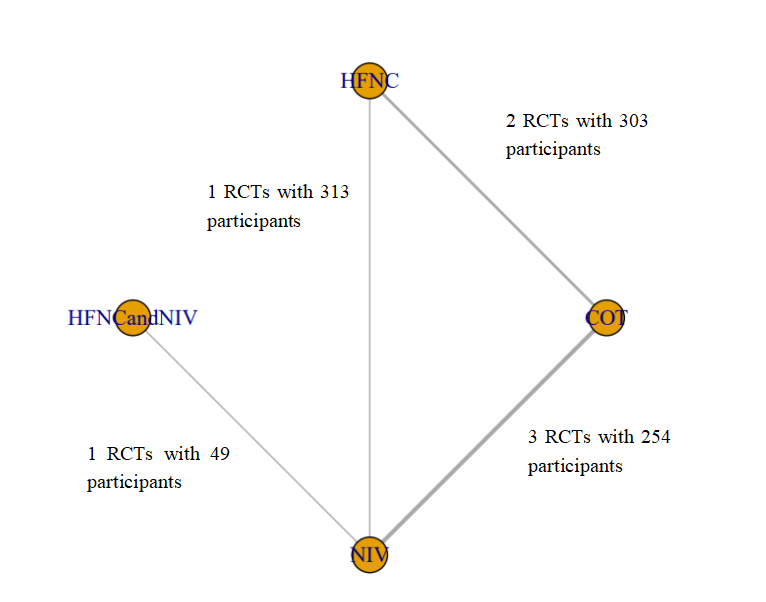
Figure S7** Network of the comparisons of ICU length of stay in the Bayesian network meta-analysis. HFNC:high-flow nasal cannula ,NIV:non-invasive ventilation ,COT:conventional oxygen therapy.

**
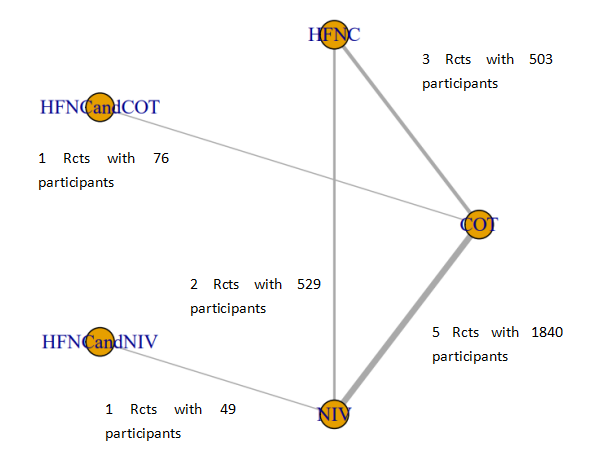
**

**Figure S8** Network of the comparisons of ICU mortality in the Bayesian network meta-analysis. HFNC:high-flow nasal cannula ,NIV:non-invasive ventilation ,COT:conventional oxygen therapy.


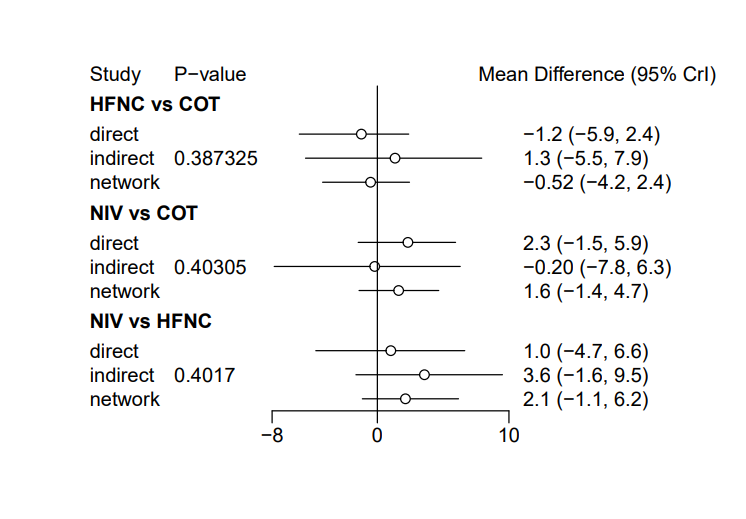


**Figure S9** A node-split analysis of ICU length of stay. HFNC:high-flow nasal cannula ,NIV:non-invasive ventilation ,COT:conventional oxygen therapy.

**
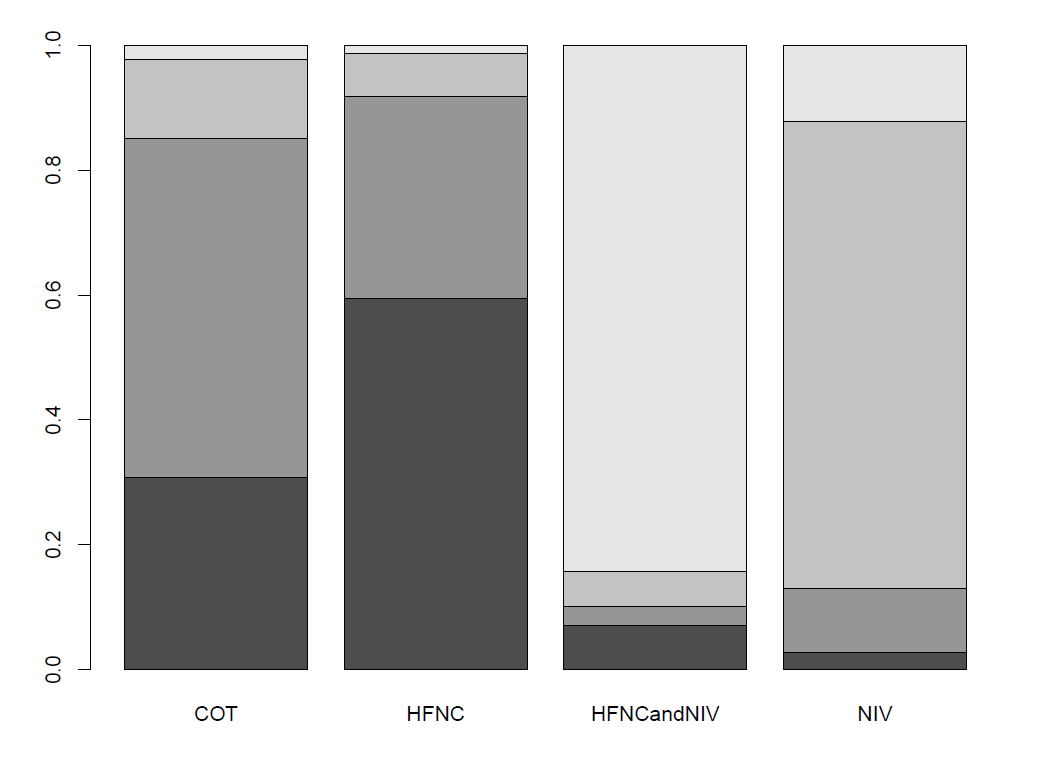
**

**Figure S10** Bar chart of the surface under the cumulative ranking curve (SUCRA) values for ICU length of stay. HFNC:high-flow nasal cannula ,NIV:non-invasive ventilation ,COT:conventional oxygen therapy.


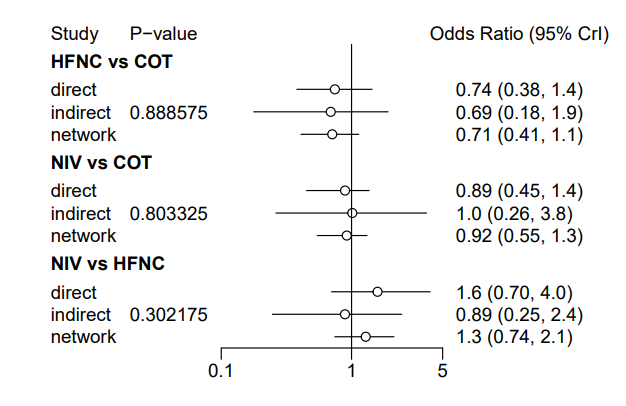


**Figure S11** A node-split analysis of ICU mortality. HFNC:high-flow nasal cannula ,NIV:non-invasive ventilation ,COT:conventional oxygen therapy.

**
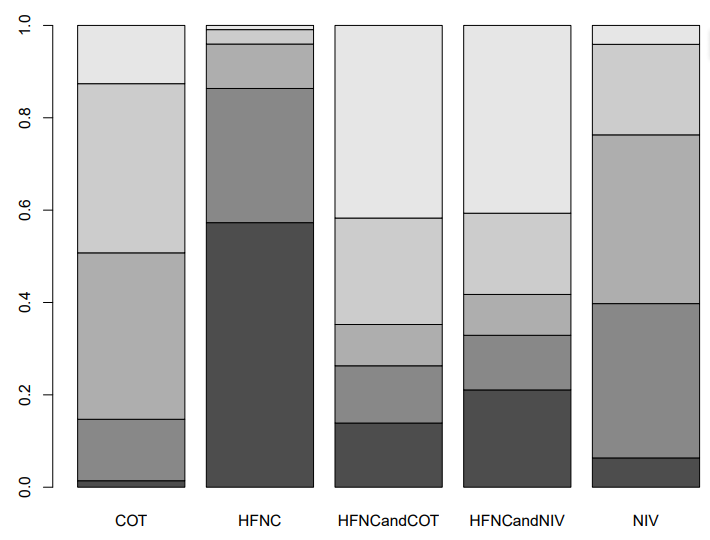
**

**Figure S12**  Bar chart of the surface under the cumulative ranking curve (SUCRA) values for ICU mortality. HFNC:high-flow nasal cannula ,NIV:non-invasive ventilation ,COT:conventional oxygen therapy.

|  | **COT** | **HFNC** | **HFNCandCOT** | **HFNCandNIV** | **NIV** |
| --- | --- | --- | --- | --- | --- |
| lowest SpO2 during intubation | 0.30% | 1.42% | **48.78%** | 46.47% | 3.04% |
| the incidence of post-intubation SpO2 <80% | 0.34% | 9.73% |  | 6.48% | **83.46%** |
| post-intubation related complications | 1.28% | **57.16%** | 26.02% | 10.76% | 4.78% |
| ICU length of stay | 30.75% | **59.41%** |  | 7.04% | 2.80% |
| ICU mortality | 1.41% | **57.29%** | 13.90% | 21.07% | 6.34% |

**Table S1** Rank probabilities for each pre-oxygenation strategy to outcome indicator.

|  | Fixed | random |
| --- | --- | --- |
| lowest SpO2 during intubation |  |  |
| Dbar | 34.65 | 13.71 |
| pD | 11.07 | 13.28 |
| DIC | 45.72 | **26.99** |
| I2 | 62% | 5% |
| the incidence of post-intubation SpO2 <80% |  |  |
| Dbar | 25.08 | 18.43 |
| pD | 12.27 | 15.97 |
| DIC | 37.35 | **34.40** |
| I2 | 32% | 8% |
| post-intubation related complications |  |  |
| Dbar | 55.61 | 21.19 |
| pD | 14.23 | 19.35 |
| DIC | 69.83 | **40.54** |
| I2 | 64% | 6% |
| ICU length of stay |  |  |
| Dbar | 15.57 | 13.99 |
| pD | 9.99 | 12.20 |
| DIC | 25.56 | 26.19 |
| I2 | 17% | **7%** |
| ICU mortality |  |  |
| Dbar | 23.52 | 22.09 |
| pD | 14.13 | 16.72 |
| DIC | 37.65 | 38.82 |
| I2 | 15% | **9%** |

**Table S2** In general，the smaller the DIC,the better the model fit is indicated. Therefore,random effect models were chosen in our NMA.
